# ERP evidence for emotion-specific congruency effects between sentences and new words with disgust and sadness connotations

**DOI:** 10.3389/fpsyg.2023.1154442

**Published:** 2023-05-12

**Authors:** Beixian Gu, Bo Liu, David Beltrán, Manuel de Vega

**Affiliations:** ^1^Institute for Language and Cognition, School of Foreign Languages, Dalian University of Technology, Dalian, China; ^2^Instituto Universitario de Neurociencia (IUNE), Universidad de La Laguna, La Laguna, Spain; ^3^School of Foreign Languages, Dalian Maritime University, Dalian, China; ^4^Psychology Department, Universidad Nacional de Educación a Distancia (UNED), Madrid, Spain

**Keywords:** disgust, sadness, pseudowords, sentences, ERPs

## Abstract

**Introduction:**

The present study investigated how new words with acquired connotations of disgust and sadness, both negatively valenced but distinctive emotions, modulate the brain dynamics in the context of emotional sentences.

**Methods:**

Participants completed a learning session in which pseudowords were repeatedly paired with faces expressing disgust and sadness. An event-related potential (ERP) session followed the next day, in which participants received the learned pseudowords (herein, new words) combined with sentences and were asked to make emotional congruency judgment.

**Results:**

Sad new words elicited larger negative waveform than disgusting new words in the 146–228 ms time window, and emotionally congruent trials showed larger positive waveform than emotionally incongruent trials in the 304–462 ms time window. Moreover, the source localization in the latter suggested that congruent trials elicited larger current densities than incongruent trials in a number of emotion-related brain structures (e.g., the orbitofrontal cortex and cingulate gyrus) and language-related brain structures (e.g., the temporal lobe and the lingual gyrus).

**Discussion:**

These results suggested that faces are an effective source for the acquisition of words’ emotional connotations, and such acquired connotations can generate semantic and emotional congruency effects in sentential contexts.

## Introduction

Language is an important vehicle to express one’s feelings and denote the emotionality of certain objects and events in people’s daily lives. During early language acquisition, children initially learn words, the smaller linguistic units, and then gradually develop their language abilities to combine words to form larger units, namely, sentences (Lightfoot, 2010). Such a general pattern also occurs with emotional language, that is, many words have an emotional valence but they are also integrated into sentences and discourses that can be governed by principles of emotional coherence (Moreno and Vázquez, 2011; Delaney-Busch and Kuperberg, 2013; Moreno and Rivera, 2014). However, the neurocognitive activities that occur when acquiring emotional connotations for words and how such connotations function in sentential contexts have been barely investigated. The present study aimed to fill that gap by measuring the brain dynamics of processing new words whose emotional connotations were acquired through an associative learning paradigm, in emotional sentential contexts. On top of that, the present study went beyond a gross categorization of “positive” and “negative,” which does not reflect the various emotions contained in words. For example, “piss” and “loneliness” are both “negative” words yet related to different emotions. Therefore, two specific emotions of disgust and sadness were selected in the present study to further tap into the various emotional nuances involved in daily verbal and written communication.

Traditionally, the role of emotion in language processing has been examined from the two dimensions of valence and arousal (Bradley and Lang, 2000). Alternatively, a discrete emotion model has been proposed, which presents more refined categorizations of emotions than merely classifying them as positive and negative valence (Ekman, 1992). In this sense, a series of studies were carried out to probe into the impact of discrete emotions on written word processing (Briesemeister et al., 2011, 2014; Silva et al., 2012; Ferré et al., 2017). For example, Briesemeister et al. (2014) reported that happy words modulated the N1 component, while general positive words affected the N400-like component, reflecting thereby a sequential processing of emotional connotation of words with discrete emotions being the basis for subsequent dimensional analysis. Such a discrete emotion effect can be interpreted through the contextual-learning hypothesis, which argues that emotion effects are based on a person’s relevant real-life experience that can be traced back to a person’s childhood (Barrett et al., 2007). Therefore, investigating the processing of words’ acquired emotional connotations from a discrete dimension could contribute to a better understanding of emotional word acquisition.

Based on this, disgust and sadness were selected as the emotions of interest in this study, which are among the basic emotions proposed by Ekman (1992). The two emotions are highly distinctive from each other, since disgust is usually triggered by external physical aversive stimuli, thus is highly relevant to human’s well-being (Rozin and Haidt, 2013) whereas sadness is more passive, internally-generated, and related to the loss of valuable goals or significant others (Kreibig et al., 2007; Lench et al., 2011). Disgust is usually accompanied by a series of typical psychophysiological responses such as nausea, reduced heart rate, amplified skin conductance, and decreased respiratory rate (Ritz et al., 2005; Stark et al., 2005). Moreover, neuroimaging studies of both brain lesion patients (Calder et al., 2000; Cantone et al., 2019) and healthy participants (Phillips et al., 1997; Wicker et al., 2003; Wright et al., 2004; Schienle et al., 2017; Viol et al., 2019) indicated that the anterior insula is the main brain structure involved in multi-modal disgust information processing. On the other hand, sadness was found to result in increased diastolic blood pressure (Sinha et al., 1992; Krumhansl, 1997) and nonspecific skin conductance (Frazier et al., 2004) and decreased pulse transit time to the ear and finger skin temperature (Gross and Levenson, 1997; Krumhansl, 1997; Kunzmann and Grühn, 2005; Kreibig et al., 2007). Moreover, converging neuroimaging study results suggested that the anterior cingulate cortex is related to processing and mediating sad experiences (Niida and Mimura, 2017; Ramirez-Mahaluf et al., 2018; Webb et al., 2018). Other negatively valenced emotions in the basic emotions such as anger and fear were ruled out for overlapping with disgust as they also tend to be triggered by threatening stimuli (Spielberger and Reheiser, 2010) despite showing different early attention modulation (Zhang et al., 2017).

An important feature of human language is that people can integrate various lexical units into a larger sentence unit (Hagoort, 2005; Hagoort and Indefrey, 2014). A series of anticipative and predictive processes take place when sentence comprehension is performed (Vosse and Kempen, 2000) both at syntactic and semantic levels. N400 is an indicator of the difficulty of integrating words into preceding contexts and the predictability of following words in unfolding sentences (Baggio and Hagoort, 2011; Kutas and Federmeier, 2011; Szewczyk and Schriefers, 2018). The N400 effects are reflected at both the semantic level and emotional level when emotional factors are involved in the sentence. Moreno and Vázquez (2011) reported reduced N400 amplitudes for highly expected negative outcomes than for highly expected positive outcomes using a passive reading paradigm while Moreno and Rivera (2014) found enhanced post-N400 frontal positivity amplitudes for unexpected positive outcomes in comparison with unexpected negative ones, indicating a bias toward possible negative results than positive ones. The N400 effects were also shown in more complex contextual setups. León et al. (2010) manipulated the emotional consistency between a story and a sentence describing the emotionality of the story and found that inconsistent emotions elicited larger N400 than consistent emotions. Positive and negative words elicited smaller N400 than neutral words following an emotional two-sentences context regardless of congruency (Delaney-Busch and Kuperberg, 2013) as well as when embedded in a pair of question and answer (Wang et al., 2013).

In addition to N400, some early ERP components have been reported to be sensitive to emotional context of words. Specifically, N1 and P2 were found to be larger for inconsistent emotions compared to consistent emotions (León et al., 2010); there was also evidence that N1 was enlarged by unexpected endings in positive-biased sentences compared to negative-biased sentences and P2 was larger for expected endings than for unexpected ones (Moreno and Rivera, 2014). Also in the early time window, Bayer et al. (2017) reported larger early posterior negativity (EPN) for negative critical words compared with positive and neutral ones in high- and low-relevance contexts. Finally, the N170 is another common component, which is usually induced by emotional words (Zhang et al., 2014) and emotional faces (Hinojosa et al., 2015), and the late positive complex (LPC) is enhanced for emotional words in sentential contexts (Delaney-Busch and Kuperberg, 2013).

In terms of the functional neuroanatomy of contextual processing of emotional words, it is difficult to disentangle activations of single word processing from those of sentence processing (Hinojosa et al., 2020). However, Mellem et al. (2016) attempted to investigate the brain structures involved in emotional sentence processing by comparing sentences with emotional–social content with meaningless sentences in a silent reading paradigm and found that the anterior superior temporal gyrus was specifically activated by emotional sentences. Moreover, Lai et al. (2015) reported activations in emotion-related brain areas such as the amygdala and insula and language-related areas such as the inferior frontal gyrus and middle temporal gyrus during the processing of sentences implying negative connotations.

The purpose of the present ERP study was twofold. Firstly, to the best of our knowledge, it explored for the first time whether new words with emotional connotations modulate brain dynamics based on their consistency with the sentential context; secondly, it investigated whether two negative but distinctive learned emotional connotations of disgust and sadness differently affect the brain dynamics of new words in sentential context. To these aims, the study was divided into a learning session and an ERP recording session. In the learning session, an associative learning paradigm used in our previous studies (Gu et al., 2021, 2022) was adopted for participants to acquire disgusting and sad connotations for pseudowords through repeatedly pairing them with emotional faces. Then the ERP session was carried out on the next day, in which all the pseudowords with acquired emotional connotations (herein new words) were paired with disgusting and sad sentences. Participants were instructed to determine the congruency between the sentence and the new words. Our hypotheses are as follows:

1. Sad new words will elicit larger EPN amplitudes than disgusting new words based on our previous study (Gu et al., 2022).
2. Incongruent sentence-new words pairs will enhance N400 compared to congruent ones as reviewed above.
3. Regarding brain sources for these effects, we expect specific emotion-related sources for disgusting and sad new words; specifically, larger activation in the insula for disgusting new words (Schienle et al., 2017; Cantone et al., 2019; Viol et al., 2019) and in the anterior cingulate cortex for sad new words (Niida and Mimura, 2017; Ramirez-Mahaluf et al., 2018; Webb et al., 2018).
4. Furthermore, given congruent sentence-new words pairs could be more deeply processed than incongruent sentence-new word pairs, we expect more robust activations in both emotion-related and language-related regions for the former than for the latter (Lai et al., 2015; Mellem et al., 2016).

## Materials and methods

### Participants

Twenty-nine Spanish college students (4 males), native speakers of Spanish, right-handed, without history of psychiatric or neurological disorders, and within an age range of 20 to 22 years old were recruited as participants for the experiment. All participants participated in the experiment voluntarily, received course credit for the learning session and money (5 euros) for the ERP recording session as reward, and provided informed consent. One participant was excluded due to excessive artifacts in the ERP data and four participants were excluded due to low response accuracies during the ERP recording session. The Research Ethics Committee of the University of La Laguna approved this study, and the experiment was conducted according to the principles expressed in the Declaration of Helsinki.

### Material

Twenty pseudowords were composed for the learning session. These pseudowords were 8–9 letters long and divided into two groups that starting with “al” and “ro” respectively. Thirty faces with disgusting and sad expressions were selected from KDEF stimuli database (Lundqvist et al., 1998). The faces selected were as follows: disgust: F02, F03, F09, F12, F13, F22, F27, F35, M12, M14, M18, M22, M24, M25, and M31; sadness: F05, F09, F11, F13, F14, F20, F22, F31, M05, M11, M13, M14, M25, M31, and M35. The hit rates of intended emotions and arousal values of the selected faces retrieved from the database documents (Karolinska Directed Emotional Faces (KDEF), 1998) are shown in Table 1. There was no significant difference in hit rates, *t*(14) = 0.429, *p = 0*.674, or arousal values, *t*(14) = 1.520, *p = 0*.151, between the two emotions. For all faces, non-facial areas (e.g., hair) were removed by applying an ellipsoidal mask. The faces were presented against a black background and with the size of 11.5 cm high by 8.5 cm wide, which equals a visual angle of 9.40° (vertical) × 6.95° (horizontal) at 70-cm viewing distance.

**Table 1:** Means of the hit rates of intended emotions (percentage) and arousal scores of the KDEF faces used in the experiment retrieved from the KDEF database.

	HIT rates (%)	Arousal
Emotion		
Disgust	92.08 (9.0)	3.65 (0.4)
Sadness	91.66 (7.0)	3.41 (0.4)
Overall	91.87 (8.0)	3.53 (0.4)

Standard deviations were shown in the parenthesis.

Other than the faces, 110 sentences expressing disgust and sadness (55 each) were composed. Two groups of students of the same college population who did not participate in the final experiment completed questionnaires to rate the emotions expressed by the sentences (39 students) and arousal values (32 students). Regarding emotion expressed, participants were instructed to first select the emotion (anger, disgust, happiness, neutral, or sadness) corresponding to the one connoted by the sentence and then to rate how confidence do they feel about their choice on a 5-point scale (1-a little sure, 5-completely sure). As for arousal, a different group of participants were instructed to rate the value in another questionnaire on a 5-point scale (1-very peaceful, 5-very exciting). No significant difference was found in terms of hit rates, *t*(54) = 1.319, *p = 0*.199, confidence scores *t*(54) = 1.037, *p* = 0.305, or arousal values, *t*(54) = 0.417, *p* = 0.683. Details of the ratings were shown in Table 2. Overall, the length of sad sentences (10.06) was longer than that of disgusting sentences (8.28).

**Table 2:** Means and standard deviations of the hit rates of emotions expressed (percentage), choice confidence scores, and arousal of the sentences used in the experiment collected through normative studies.

	HIT rates (%)	Choice confidence scores	Arousal
Emotion			
Disgust	89.70 (8.0)	4.71 (0.3)	4.32 (0.4)
Sadness	86.81 (9.0)	4.66 (0.3)	4.25 (0.7)
Overall	88.26 (9.0)	4.67 (0.3)	4.29 (0.6)

### Learning session

Following the procedures implemented by Gu et al. (2022), the learning session consisted of a training phase, an evaluation phase, and a generalization phase. During the training phase, each trial began with a fixation cross at the center of the screen for 1,000 ms, followed by a sole presentation of the face of 1,000 ms, and then the pseudoword presented in Times New Roman font and size 50 on top of the face for 5,000 ms. There were in total three training blocks in the training phase. In the first block, there were four face—pseudoword pairs and in the second and third blocks, there were three. Each pair within the training block was repeated six times, which resulted in 48, 36, and 36 trials in each of the three blocks, respectively. The pairing between faces and pseudowords was counterbalanced across participants. After each training block, there was a pseudoword selection test in which participants were instructed to select which of two presented pseudowords matched the face presented. Each selection trial began with a fixation cross at the center of the screen for 1,000 ms, followed by one of the faces presented in the training block for 1,000 ms, and then two pseudowords from the same training block appeared at the lower-left corner of the screen for 5,000 ms in Time New Roman font and size 18. Participants made their selections by pressing “1” on the keyboard for the pseudoword on the left and “2” for the one on the right (not the keypad) with their left hands. There were 30 test trials in total, 10 after each learning block.

After participants completed all three training blocks and pseudoword selection tests, they proceeded to the evaluation phase, consisting of a face-pseudoword matching test. There were 20 trials in this phase and the presentation sequence was as follows: a fixation cross at the center of the screen for 1,000 ms—a face from the previous three learning blocks for 1,000 ms—a pseudoword from the previous three learning blocks on top of the face along with two options of “correct” and “incorrect” for 5,000 ms at the lower-left corner of the screen. Like in the pseudoword selection test, participants were instructed to make their selections by pressing “1” on the keyboard for “correct” and “2” for “incorrect.” The fonts and sizes of the pseudowords and “correct” and “incorrect” options were identical to those in the pseudoword selection test.

**Figure 1:**
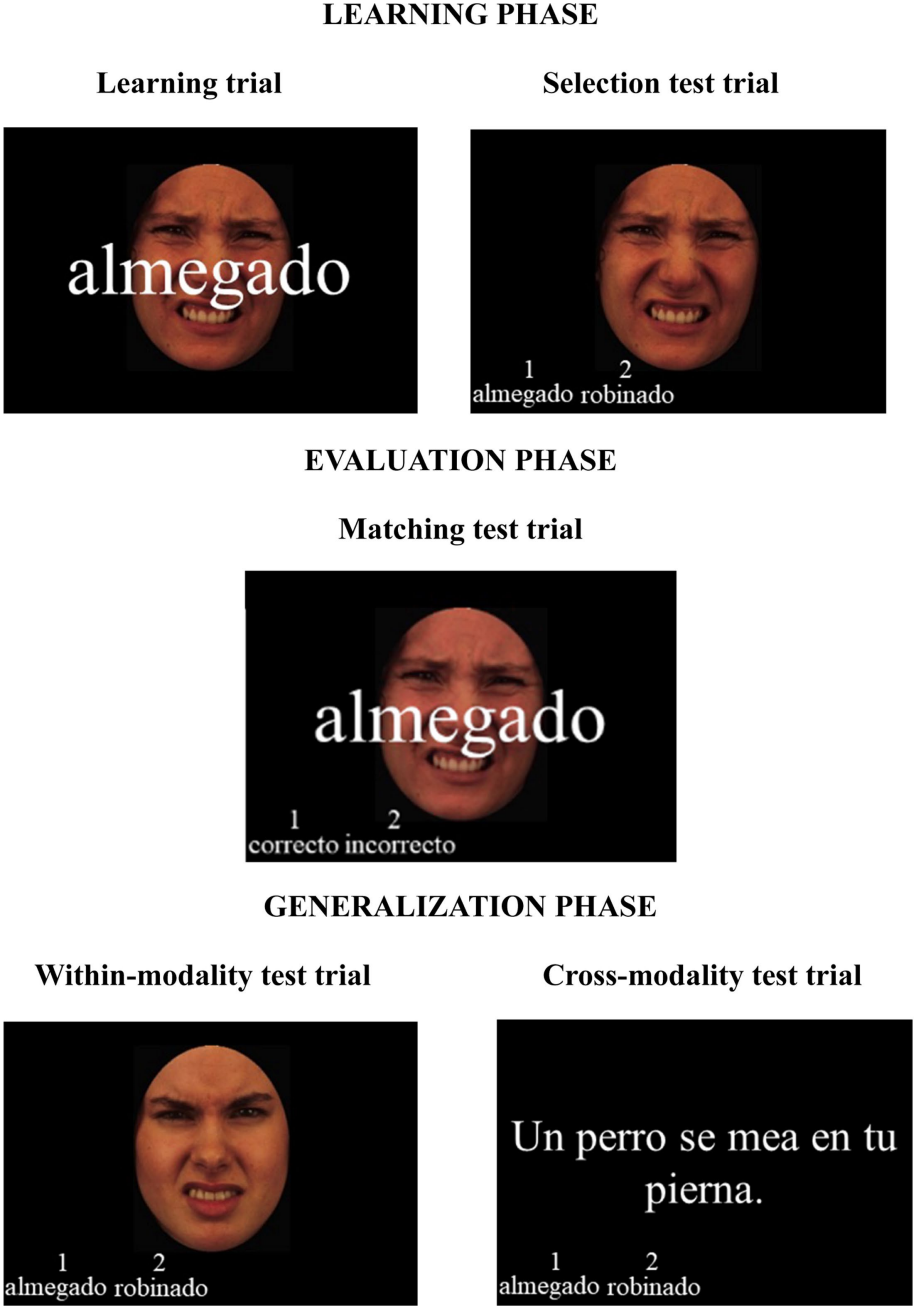
Learning session. Illustration of the last frame of trials employed in the learning phase, the evaluation phase, and the generalization phase. The procedures are explained in the text and in Gu et al. (2022). The English translation of the sentence in the figure is “A dog pisses on your leg”. The faces used were extracted from the KDEF stimuli database (Lundqvist et al., 1998).

Finally, there was a generalization phase, which included two tests. The purpose of these two tests was to probe whether participants have acquired the emotions, rather than specific faces, during the training phase. The first test in this phase was a within-modality generalization test. The presentation sequence of the 10 trials was the same as the pseudoword selection test only the faces were replaced with a new set of faces that also express either disgust or sadness. Participants were instructed to select the pseudoword that best describes the emotion expressed by the new face among the two options by pressing “1” or “2” on the keyboard just like in the pseudoword selection test. The second test was a cross-modality generalization test. In this test, sentences expressing disgust (e.g., Un perro se mea en tu pierna / A dog pees on your leg) or sadness (e.g., Tus padres se divorciaron / Your parents divorced) were introduced as probe materials. There were 10 trials in this test and the sentences used here never appeared in the ERP recording session. Each trial began with a fixation cross for 1,000 ms, followed by a sentence at the center of the screen for 1,000 ms in Times New Roman font and size 30, and then two pseudowords from the previous learning session were presented at the lower-left corner of the screen until participants made a response. Participants made their selections in the same way as previous, pressing “1” or “2” on the keyboard. Participants received immediate feedback after response in all the tests throughout the three phases indicating whether their selections were correct or not. The Figure 1 illustrates the three phases, depicting the last frame of the trials.

### ERP recording session

**Figure 2:**
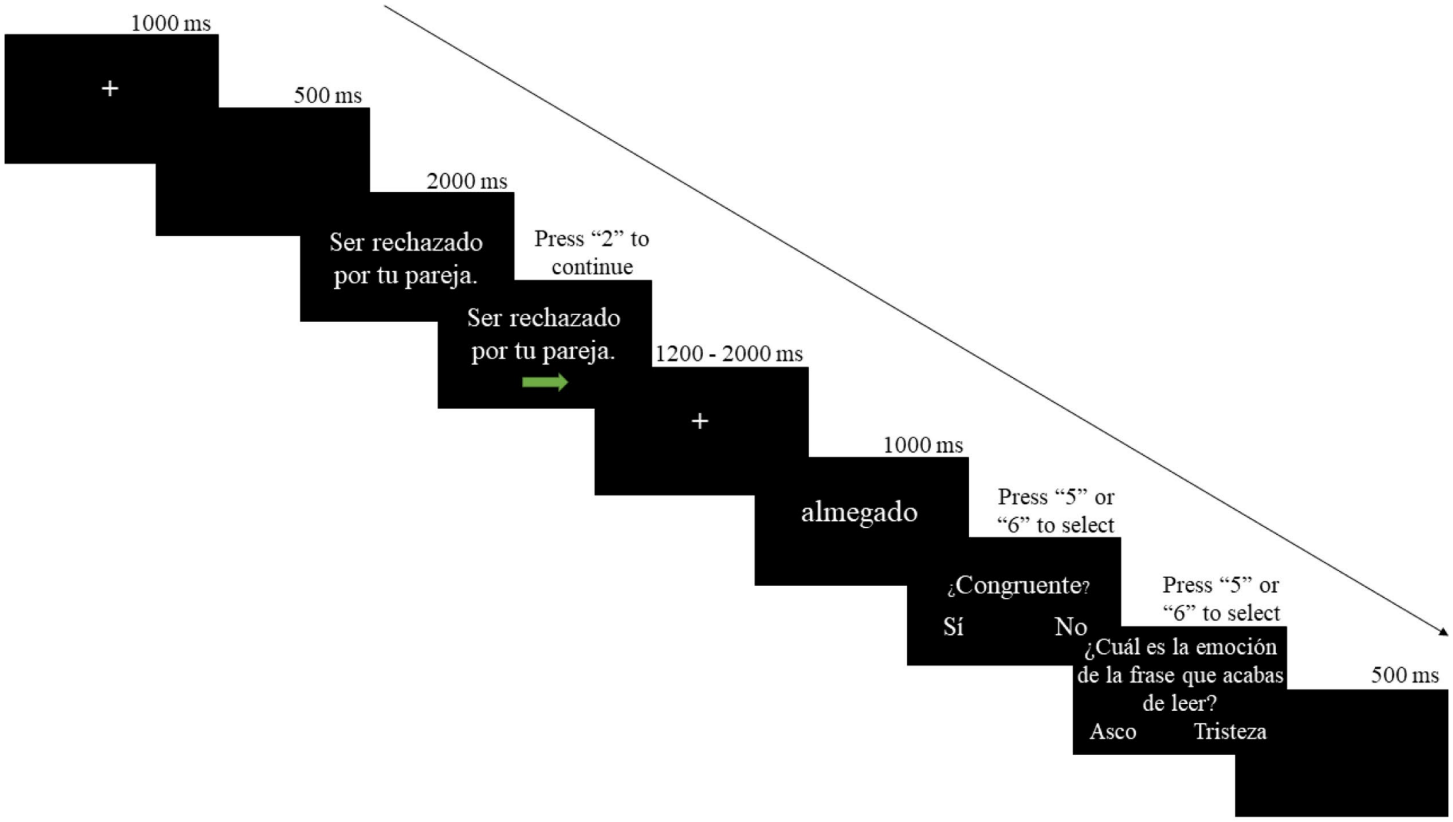
Outline of an experimental trial in the ERP recording session. The English translation of the sentence in the figure is “Being rejected by your partner” and the question is “What is the emotion of the sentence you just read?”.

Participants came to the ERP recording session on the next day after completing the learning session. Upon arrival, participants first completed a refreshing session in which they went through all four tests in the preceding learning session. Participants had to reach at least 85% accuracy to be qualified for the ERP session. Such a design enabled us to investigate the effect of acquired emotional connotations rather than short-term memory and excluded the impact of tiredness due to long experimental duration. Next, they were seated comfortably in a chair in a sound-attenuated room. Experimental trials were presented on a computer screen using E-Prime 2.0 at approximately 80 cm from the participants. During the ERP recording session, participants used a joystick for responses. Each trial started with a fixation cross presented at the center of the screen for 1,000 ms, followed by a 500 ms blank screen. Next, a sentence was presented at the center of the screen in Times New Roman font and size 20 and a green arrow appeared under the sentence after 2,000 ms. At this moment, participants could press “2” on the joystick autonomously to proceed if they finished reading the sentence. A fixation cross was presented again at the center of the screen for a random interval between 1,200 and 2,000 ms and then the new word for 1,000 ms (Times New Roman fonts, size 50). Then the word “¿Congruente?” (Congruent?) along with two options of “Sí” (Yes) and “No” were presented in Times New Roman font and size 20, and the participants judged whether the emotional connotations between the sentence and the pseudoword were congruent. Participants were instructed to press “5” and “6” on the joystick to respond. The mapping between joystick buttons and options was counterbalanced across participants. For 20% of the experimental trials, there was an additional emotion probe test followed the congruency test with the sentence “¿Cuál es la emoción de la frase que acabas de leer?” (What is the emotion of the sentence you just read?) presented at the center of the screen to ensure that the participants were reading the sentence stimuli carefully. Under the sentence, there were two options of “Asco” (Disgust) and “Tristeza” (Sadness). Participants were instructed to select by pressing “5” or “6” on the joystick. Once again, the mapping between the buttons and options was counterbalanced across participants. Each experimental trial ended with a blank screen for 500 ms and then continue to the next one. There were two blocks of 100 sentences in the experimental session. Sentences followed by a disgusting pseudoword in block 1 were followed by a sad pseudoword in block 2, and vice versa. See Figure 2 for an example of experimental trials.

## EEG recording and analysis

Electroencephalography (EEG) and electrooculography (EOG) data were recorded with Ag/AgCl electrodes mounted in elastic Quick-caps (Compumedics). EOG data were recorded from two bipolar channels: one from two electrodes placed at the outer canthus of each eye and the other from two electrodes above and below the left eye. EEG data were recorded from 60 electrodes arranged in accordance with the standard 10–20 system with additional electrodes placed on the left and right mastoids (M1 & M2). All electrodes were re-referenced offline to the left and right mastoids. EEG and EOG signals were amplified at 500 Hz sampling rate using Synamp2 amplifier (Neuroscan; Compumedics), with high- and low-pass filters set at 0.05 and 50 Hz, respectively. The impedances of the electrodes were kept at under 5 k Ω. The ERP recordings were time-locked to the onset of each pseudoword and ERP analysis was applied to an epoch extending from 200 ms before to 1,000 ms after the pseudoword onset. EEG preprocessing and analysis were performed with Brainstorm (Gramfort et al., 2010). Drifting, ocular, and motor artifacts in experimental trials were rejected by visual inspections before analysis. Eye blinks and movements were removed by applying the Independent Component Analysis (ICA; Lee, 1998). Finally, trials with EEG voltages exceeding 70 μV measured from peak to peak at any channel were removed (12%).

After preprocessing, the ERP data were computed and analyzed using Brainstorm. Baseline was set at 200 ms before stimuli onset. ERP waveforms were obtained by averaging baseline-corrected EEG segments. The obtained ERP waveforms were then statistically analyzed using the cluster-based random permutation method implemented in Fieldtrip (Maris and Oostenveld, 2007), which conducts multiple spatiotemporal comparisons by identifying clusters of significant differences between conditions (sample points in close spatial and temporal proximity) over the whole ERP segment (32 sample points: 500 time points, from 200 ms prior to and 1,000 ms after pseudoword onset, and 64 channels) so as to find components and time windows of interest. Given that only pair-wise comparisons are applicable to this method, experimental conditions were collapsed based on the main effects of emotion (disgust & sadness) and congruency (congruent & incongruent). To further explore the interaction effect, the pair-wise comparison was done between the differences of the two congruency conditions of each emotion (disgust-congruent minus disgust-incongruent & sadness-congruent minus sadness incongruent). ROIs were created for sets of neighboring solution points showing significant differences (*p* < 0.05) and 2*2 (disgust & sadness * congruent & incongruent)” here to further demonstrate how the ANOVAs were performed using the mean absolute values of current densities in those ROIs and time windows.

## Source localization analysis

Source localization was carried out using Brainstorm and EEG data were co-registered with a standard anatomical template (ICBM152) and boundary element head models were constructed with OpenMEEG (Pascual-Marqui, 2002). Source activities were estimated for each condition using sLORETA (Pascual-Marqui, 2002) with unconstrained source orientations. Differences between every two conditions were calculated for each participant and ROIs were created accordingly for sets of neighboring solution points (no less than 30) showing significant differences (*p* < 0.01). Mean absolute values of the current densities of the ROIs were then exported for running pairwise t-tests as in the EEG data analysis.

## Results

### Behavioral results

#### Learning session

Participants’ learning effects were demonstrated through the two tests in the generalization phase since they could best reflect the acquisition of emotional connotations for the pseudowords.

The mean response accuracy for disgusting and sad trials in the two tests was shown in Table 3. There was no significant difference regarding response accuracy of the two types of trials in both the within-modality (*t*(23) = 0.514, *p* = 0.612) and the cross-modality [*t*(23) = 2.000, *p* = 0.057] generalization tests.

**Table 3:** Means and standard deviations of response accuracy (percentage of correct choices) in the within- and the cross-modality generalization tests.

	Within-modality (%)	Cross-modality (%)
Emotion		
Disgust	89.17 (12.0)	95.83(8.0)
Sadness	86.67 (22.0)	89.17(16.0)
Overall	87.92 (17.0)	92.50(12.0)

### ERP recording session

The mean response accuracies for the congruency and emotion choice tasks in the ERP recording session were 96.85% (SD = 3.67%) and 97.71% (SD = 5.10%) respectively, indicating that the participants have fully understood the experimental instructions and paid attention to the stimuli throughout the experimental process.

### ERP results

**Figure 3:**
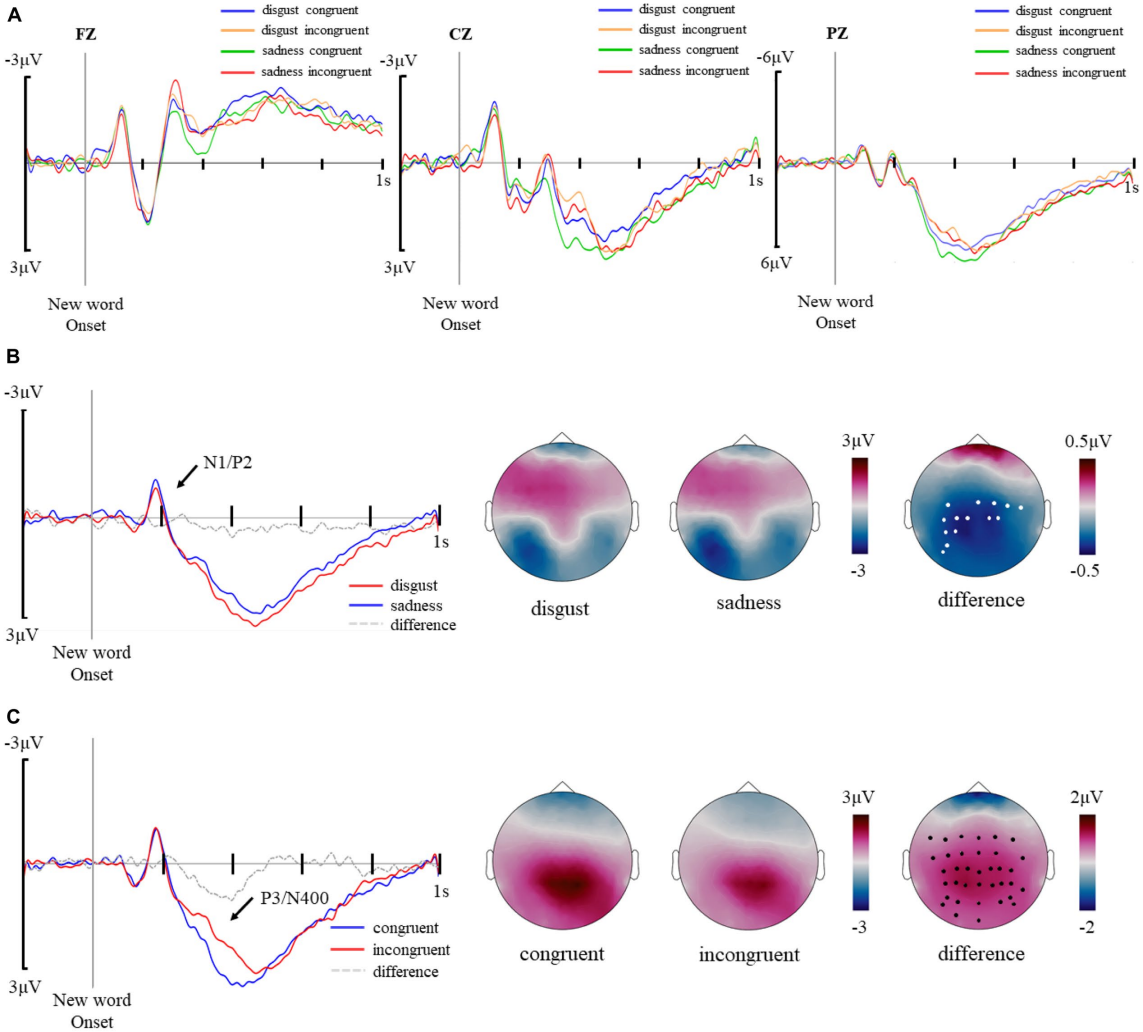
**(A)** Average waveforms of disgust congruent, disgust incongruent, sad congruent, and sad incongruent trials in a cluster of representative electrodes (FZ, CZ, PZ). **(B)** The main effect of emotion. Average waveforms of disgusting new words and sad new words in a cluster of electrodes showing significant differences (C3, CP5, CP3, CP1, P5, P3, PO5, PO7, CZ, C2, C4, C6, CP2, and CP4) and topographical distributions of the central-parietal negativity for disgusting and sad new words, and for their difference. Electrodes showing significant differences were marked with white dots. **(C)** The main effect of congruency. Average waveforms of congruent trials and incongruent trials in an extensive cluster, which includes fronto-central and parietal electrodes showing significant differences (FC5, FC3, FC1, FCZ, FC2, FC4, C5, C3, C1, CZ, C2, C6, CP5, CP3, CP1, CPZ, CP2, CP4, CP6, P5, P3, P1, PZ, P2, P4, P6, P8, PO7, PO5, PO3, POZ, PO4, PO6, PO8, O1, and OZ) and topographical distributions of the central-parietal positivity for congruent trials, incongruent trials, and differences between the two types of trials. Electrodes showing significant differences were marked with black dots.

The main effects of emotion and congruency were analyzed separately. The waveforms of the four experimental conditions were presented in Figure 3A. For the emotion analyses, congruent and incongruent trials were collapsed and the cluster-based random permutation method was adopted to locate significant clusters in the time window of 0–1,000 ms by comparing between disgusting trials and sad trials. For the congruency analyses, the two emotion conditions were collapsed and congruent and incongruent trials were compared. For the interaction analyses, the differences of the two congruency conditions of each emotion were compared. With such an approach, two clusters were identified that reflect the main effect of emotion and the main effect of congruency respectively, with the former extending from 146–228 ms, coincident with a N1/P2 complex (Figure 3B), and the latter from 304–462 ms, coincident with partially overlapped P3 and N400 components (Figure 3C). Meanwhile, no significant effect was spotted in the interaction analyses.

#### The main effect of emotion (146–228 ms)

The waveform and topography of the main effect of emotion are illustrated in Figure 3. Sad new words elicited more negative amplitudes than disgusting new words in the central-parietal area in the 146–228 ms time window, *t*(23) = 2.904, *p* = 0.008. Repeated ANOVA analyses revealed an interaction effect of emotion*congruency, *F*(1, 23) = 8.377, *p* = 0.008, *η*^2^_p_ = 0.267, with sad congruent trials eliciting larger negative amplitudes than disgusting congruent trials, *t*(23) = 3.094, *p* = 0.02.

#### The main effect of congruency (304–462 ms)

The waveform and topography of the main effect of congruency are illustrated in Figure 3. Congruent trials elicited larger positive amplitude than incongruent trials in the central-parietal area in the 304–462 time window, *t*(23) = 4.872, *p* < 0.001. Repeated ANOVA analyses revealed a main effect of emotion, *F*(1, 23) = 7.621, *p* = 0.011, *η*^2^_p_ = 0.249, and a main effect of congruency, *F*(1, 23) = 23.764, *p* < 0.001, *η*^2^_p_ = 0.508, with sad congruent trials eliciting larger positive amplitudes than sad incongruent trials, *t*(23) = 4.123, *p* < 0.001, and disgusting incongruent trials, *t*(23) = 5.465, *p* < 0.001, and disgusting congruent trials eliciting larger positive amplitudes than disgusting incongruent trials, *t*(23) = 2.847, *p* = 0.039.

## Source localization

**Figure 4:**
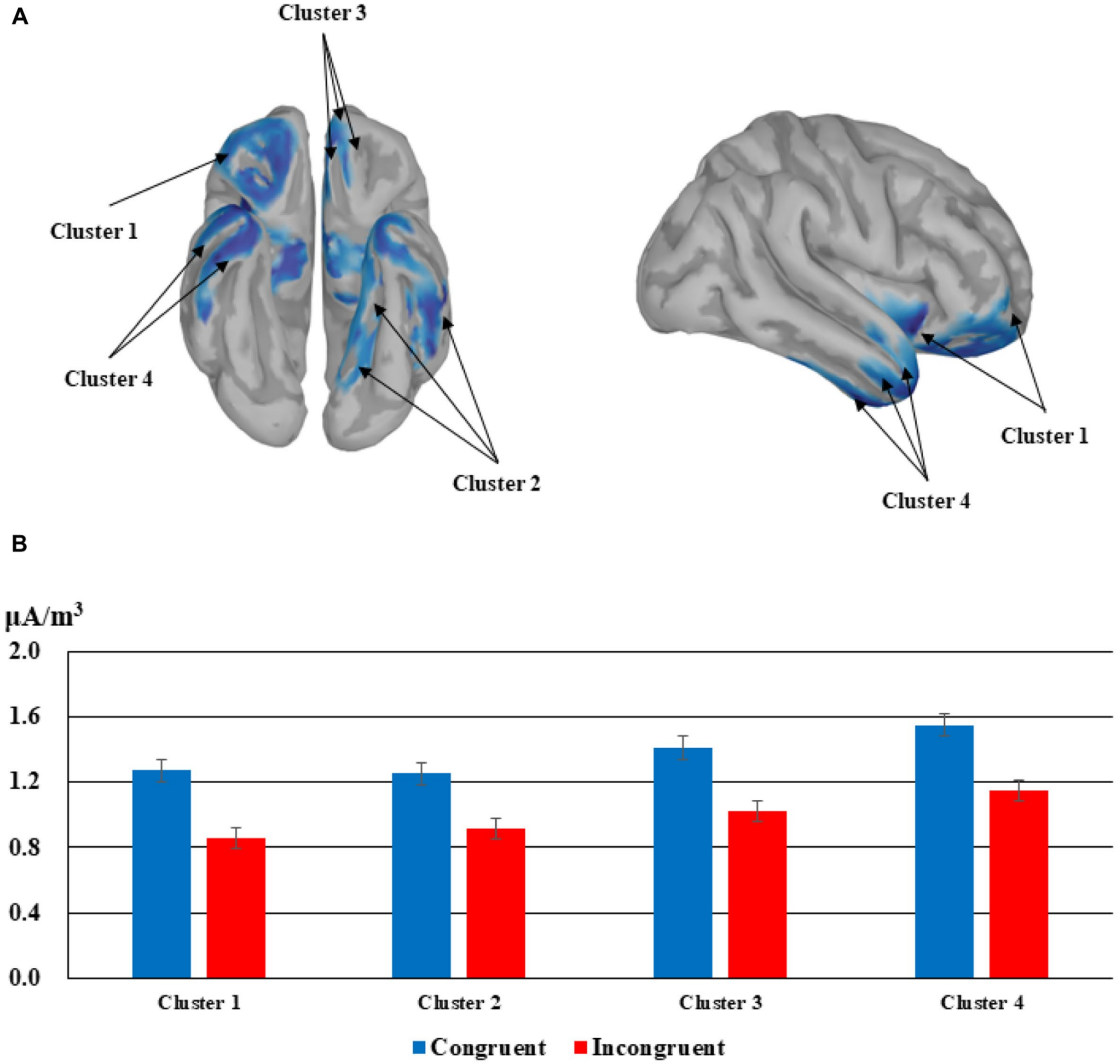
Source localization for the congruency effects corresponding to the ERPs in the time window of 304–462 ms. **(A)** The cortices demonstrating significant effects in four clusters (Cluster 1: lateral orbitofrontal cortex & insular cortex & rostral middle frontal gyrus; Cluster 2: the left inferior temporal lobe & parahippocampal gyrus & lingual gyrus; Cluster 3: the rostral anterior cingulate cortex & medial orbitofrontal cortex & lateral orbitofrontal cortex; Cluster 4: the right superior temporal gyrus & middle temporal gyrus & inferior temporal gyrus). **(B)** Mean current densities for congruent trials and incongruent trials in the four clusters.

The source localization did not reveal any converging result for the main effect of emotion but four clusters were identified for the main effect of congruency. Congruent trials elicited larger current densities than incongruent trials in the four significant clusters, that is the lateral orbitofrontal cortex & insular cortex & rostral middle frontal gyrus, *t*(23) = 4.772, *p* < 0.001, the left inferior temporal lobe & parahippocampal gyrus & lingual gyrus, *t*(23) = 3.801, *p* < 0.001, the rostral anterior cingulate cortex & medial orbitofrontal cortex & lateral orbitofrontal cortex, *t*(23) = 4.292, *p* < 0.001, and the right superior temporal gyrus & middle temporal gyrus & inferior temporal gyrus, *t*(23) = 4.158, *p* < 0.001. See Figure 4 for details.

## Discussion

The present study examined how acquired disgusting and sad connotations affect written word processing under sentential contexts. Participants acquired the two emotional connotations for new words by pairing pseudowords with emotional faces in a learning session and the high accuracies in responding to the tests indicated that the acquisition was successful. In the ERP recording session on the next day, participants were presented with sentential contexts expressing either disgusting or sad events followed by one of the new words learned in the previous session. The instruction to participants was to judge the emotional congruency between the context and the new word. The results revealed several main facts. Firstly, sad new words elicited larger negativity than disgusting new words in the early time window, coincident with N1/P2 components complex, indicating that the emotional learning succeeded to show differential modulation of a brain signature between new words with two discrete negative emotions. Secondly, new words that were emotionally congruent with the sentence induced larger positive-going waveform than those that were emotionally incongruent with the sentence in a later time window (304–462 ms), coincident with a N400 and P3 components. Thirdly, the source localization for this congruency effect revealed activations mainly in fronto-medial and temporal brain structures, which further demonstrates successful emotional connotation acquisition for the new words and a congruency effect of emotion.

### Behavioral and ERP results

These results were possible because the methodology used in the learning session (see also Gu et al., 2021, 2022). Participants achieved high response accuracy in the within- and cross-modality tests, which suggests that rather than merely associating the new words with specific faces, they acquired the corresponding emotions expressed by those faces and were able to apply the new words to refer to new materials. We intentionally used a categorical design of new words (first two letters being “al” and “ro”) to reduce the learning difficulty as the learning session was relatively short. Such a design was employed in our previous study as well (Gu et al., 2021, 2022). The possibility that participants only pay attention to the first two letters could be ruled out since evidence suggests that lexical processing is not conducted in a letter-by-letter fashion (Shallice and Saffran, 1986). Meanwhile, such a design emulates the semantic structure of real emotional words that are often organized in morphological sets with the same root (e.g., disgust, disgusting, disgusted).

ERP data analysis revealed a main effect of emotion between 146 and 228 ms, represented by larger centro-parietal negativity elicited by sad new words than disgusting new words combined with a positivity distributed in the frontal-central area, which resembles the N1/P2 complex (Begleiter et al., 1979; Scott et al., 2009). Such early components were reported in previous studies focusing on emotion and words to be reflecting the effect of emotion on early lexical access (Sereno et al., 1998; Sereno and Rayner, 2003; Hofmann et al., 2009) and attention allocation (Kanske and Kotz, 2007; Gu et al., 2022). More importantly, such early components suggest different processing patterns of specific emotions (Thomas et al., 2007; Mattavelli et al., 2016). The pattern discovered here is contrary to our previous study investigating individual emotional new word processing (Gu et al., 2022), in which disgusting new words elicited larger EPN amplitudes than sad new words. The difference could be attributed to the influence of emotional sentential contexts that either reduce the attentional resource costs or decoding difficulty of disgusting new words, whose connotations were acquired through faces (Suzuki and Akiyama, 2013; Leleu et al., 2015). In addition, ERP components were highly sensitive to different experimental designs and materials, for example, Scott et al. reported enlarged N1 amplitudes for isolated negative words than for positive words (2009) while Moreno and Rivera found a more positive-biased N1 in their study involving sentential contexts (2014).

The main effect of congruency was shown through a positive/negative complex from 304 to 462 ms, which was similar to the P3/N400 complex given its time window and scalp distribution (Chwilla et al., 1995). According to previous studies, P3 could be closely related to decision-making (O’Connell et al., 2012; Kelly and O’Connell, 2013; Twomey et al., 2015) as well as event categorization (Kok, 2001; Polich, 2007) processes, which are in line with the characteristics of the task used in our ERP recording session, where the participants were instructed to determine the emotional congruency between the new word and the preceding context. Other than that, the complex could be interpreted as a semantic N400 effect that is commonly reported in processing emotional words in contexts (for review, see Hinojosa et al., 2020). The waveform was more negative-going for incongruent trials than for congruent trials, which reflects higher integration difficulties and/or lower anticipation possibilities for emotionally incongruent new words (Baggio and Hagoort, 2011; Kutas and Federmeier, 2011; Szewczyk and Schriefers, 2018). The N400 effect thus reflected the successful acquisition of emotional connotation through emotional faces in the learning session. However, such an interpretation should be viewed with caution as it was difficult to disentangle the N400-like negativity from the P3-like positivity, which overlap in the same time window. Nevertheless, the P3/N400 complex spotted here was induced by the emotional congruency at the sentence level rather than pure associative pairing between faces and new words.

### Source localization results

The role of emotion in the congruency judgment process was further verified through source localization results. Congruent trials induced larger current densities in four different ROIs related to emotion and language processing than incongruent trials during the 304–462 ms time window. The first group of structures includes the lateral orbitofrontal cortex, the insular cortex, and rostral middle frontal gyrus, which were reported to be related to emotion regulation (Gross, 2002; Hooker and Knight, 2010), disgust processing (Calder et al., 2000; Krolak-Salmon et al., 2003; Wright et al., 2004), and emotion generation (Waugh et al., 2010, 2014; Roy et al., 2012), respectively. Next, two word recognition related structures, left inferior temporal lobe (Sharp et al., 2004; Antonucci et al., 2008; Trimmel et al., 2018) and lingual gyrus (Mechelh et al., 2000; Xiao et al., 2005; Ghosh et al., 2010), were activated more for congruent trials than for incongruent ones, and the same pattern applies to parahippocampal gyrus, a structure involved in emotion identification and regulation (Almeida et al., 2009; Zhu et al., 2019). In the third ROI, the rostral anterior cingulate cortex is the neural locus for sad information processing as suggested recently (Niida and Mimura, 2017; Ramirez-Mahaluf et al., 2018; Webb et al., 2018). Similarly, the orbitofrontal cortex was reported to be related to depression and emotion regulation (Rolls et al., 2020), despite the medial section is more closely associated to negative emotions than the lateral section (Northoff et al., 2000). The temporal lobe in the fourth ROI is another brain structure demonstrated to be critical to emotion processing (Adolphs, 2002; Monti and Meletti, 2015). Specifically, the superior temporal gyrus is involved in emotion perception of facial stimuli (Bigler et al., 2007; Radua et al., 2010), the middle temporal gyrus is connected with face recognition (Pourtois et al., 2005) and word meaning retrieval (Acheson and Hagoort, 2013), and the inferior temporal gyrus is in support of word recognition (Nobre et al., 1994). To sum up, the above activation patterns suggested that firstly, the emotional connotation acquisition for the new words was successful and effective, particularly reflected through the larger activations in the insula and anterior cingulate cortex; secondly, the congruency effect was emotional as new words that are emotionally congruent with the preceding contexts induced larger current densities in emotion- and word recognition-related regions.

### Implications and possible future research directions

One of the novelty in the present experimental design was employing emotional faces rather than emotional scenes commonly used in previous studies (Fritsch and Kuchinke, 2013; Kuchinke et al., 2015), as associative contexts in the learning session. This method succeeded in showing that disgusting new words and sad new words were processed differently, as reflected the modulation of the N1/P2 complex in the early time window. Moreover, this study revealed that emotionally constraining contexts influence the processing of incoming emotional information (Hinojosa et al., 2020), even recently acquired as was the case here. Importantly, this study provided evidence from a discrete emotion model, ruling out the impact of arousal as that of the associative contexts and experimental contexts was matched. Finally, this study presented clues relevant for second language (L2) acquisition of words emotional connotations. It suggests that pairing L2 words with facial expressions (of teachers and/or students) could be a simple and efficient method to induce emotional semantics of L2 in classroom learning. Furthermore, it supports the idea that embodied representations of emotional words contribute to reinforce meaning (Pulvermüller, 2013).

Meanwhile, future studies could conduct similar investigations on a more gender-balanced participant group to compare the learning and processing of words’ emotional connotations between males and females, since gender has been reported to affect the ERP amplitudes (Yang et al., 2016) and latency (Toufan et al., 2021) as well as emotional cognition (Lin et al., 2021). In addition, neutrality could be introduced as a research baseline to further demonstrate the differential processing of words’ acquired emotional connotations in sentential contexts.

## Conclusion

The current study presents evidence of how words’ acquired disgusting and sad connotations are processed in emotionally congruent and incongruent sentential contexts. ERP results suggested that sad new words elicited larger negativity compared with disgusting new words, which demonstrates unique brain temporal dynamics for processing the two discrete emotional connotations acquired through emotional faces in emotional contexts. Meanwhile, an emotional congruency effect was revealed by enhanced positivity for emotionally congruent trials than incongruent trials. In addition, the congruency effect was accompanied by larger activations in emotion regulation and processing-related brain structures for congruent trials, which verifies the emotion acquisition effect and highlights the role of emotion in the congruency judgment process. In conclusion, the present study adds relevant evidence in the field of emotional words processing by demonstrating the brain dynamics during the processing of words’ acquired emotional connotation as well as their sensitivity to sentential context.

## Data availability statement

The raw data supporting the conclusions of this article will be made available by the authors, without undue reservation.

## Ethics statement

The studies involving human participants were reviewed and approved by The Research Ethics Committee of the University of La Laguna. The patients/participants provided their written informed consent to participate in this study.

## Author contributions

BG: experiment design and conduction, data processing, and paper writing. BL: experiment design and conduction and data processing. DB: experiment design, data processing, and paper writing guidance. MV: experiment design and paper writing guidance. All authors contributed to the article and approved the submitted version.

## Funding

European Regional Development Fund (Grant RTI2018-098730-B-I00): EEG equipment and facilities, and participants’ rewards. The National Social Science Fund of China (22CYY019): Open access publication fees. The Fundamental Research Funds for the Central Universities (DUT21RC(3)094): Information retrieval and data processing equipment procurement. The Research Funds for the School of International Education at Dalian University of Technology (SIE21RYB12): Information retrieval and data processing equipment procurement.

## Conflict of interest

The authors declare that the research was conducted in the absence of any commercial or financial relationships that could be construed as a potential conflict of interest.

## Publisher’s note

All claims expressed in this article are solely those of the authors and do not necessarily represent those of their affiliated organizations, or those of the publisher, the editors and the reviewers. Any product that may be evaluated in this article, or claim that may be made by its manufacturer, is not guaranteed or endorsed by the publisher.
