# Supplementary material for: ERP evidence for emotion-specific congruency effects between sentences and new words with disgust and sadness connotations
Source: Front Psychol. 2023 May 12;14:1154442. doi: 10.3389/fpsyg.2023.1154442 (PMC10213552; doi:10.3389/fpsyg.2023.1154442)
Supplement: Supplementary file 1 [file Table_1.DOCX]

Pseudowords:

| aljuvado | robinado |
| --- | --- |
| almegado | robicado |
| alfirdado | rofeludo |
| alsastado | ronagado |
| altorzado | roninado |
| altapido | ronicado |
| alsudido | roranado |
| algifado | rogimado |
| alfusado | rofenado |
| alyapado | royafado |

Disgusting Sentences:

El olor de un vater sucio y atascado.

El olor corporal de una persona sudorosa.

Encontrarte basura podrida en la cocina.

Lavar los platos mohosos con restos de comida.

Rascarse el trasero y olerse el dedo.

Escarbarse la nariz y limpiar el dedo en la cortina.

A tu vecino del tranvía le huelen los pies sudorosos.

Ver a un perro comiendo su propio excremento.

Sin saber lo que era te bebes un trago de sangre de vaca.

El olor de axilas sudorosas de alguien en la guagua.

Notar un fuerte olor a pedo en un lugar público.

Ver a una persona vomitar cerca de ti.

Encontrar una cucaracha muerta en la salchicha.

Caer sentado sobre una caca de perro.

Encontrar un ratón muerto en tu plato de queso.

Encontrar una rana muerta en tu lata de coca.

Encontrar una uña en tu helado de vainilla.

Desatascar el inodoro con las manos desnudas.

Notar el agua del inodoro que te salpica sobre la piel.

Encontrarte gusanos vivos en la ensalada.

Ver varias moscas revoloteando sobre en el fregadero.

Ver un pájaro muerto cubierto de moscas verdes.

Limpiar una alcantarilla atascada con el agua a la cintura.

El olor a huevo podrido en la nevera apagada.

Ver una pizza caducada llena de gusanos.

Encontrar una tirita en las papas fritas.

Ver una herida supurando pus maloliente.

Ves a un hombre borracho tendido en su vómito.

Notas el olor a ropa sudada en medio de la multitud.

Notas olor del contenedor de basura.

Encuentras una polilla en tu café.

Te sientas en un baño público sin tapa.

Ves a un hombre escupir carne masticada.

Limpias los pelos en el desagüe del baño.

Usaste accidentalmente el cepillo de dientes de otro.

La bolsa de basura está goteando líquido.

Pisas con los pies descalzos el suelo grasiento.

Te limpias la nariz con un pañuelo sucio.

Buscas un reloj perdido en la bolsa de basura.

Ves gusanos blancos retorciéndose en la basura.

Al pasar junto a la pescadería hueles a pescado podrido.

Sientes una cucaracha caminando en tu brazo.

Ves moscas flotando en tu sopa.

Sacas las tripas del pescado con tus manos.

Sin darte cuenta te comes una chuleta podrida.

No hay agua en la cisterna y usas un váter sucio.

Das un mordisco a la fruta y te comes un gusano.

Dar un mordisco a un melocotón podrido.

Encontrarte en la calle una rata destripada por un coche.

Ver al carnicero extrae los intestinos de una vaca muerta.

Sad Sentences:

Vivir muy de cerca el Alzheimer de tu abuelo.

Romper con tu pareja después de muchos años juntos.

Comprobar la situación de pobreza en la que han caído tus vecinos.

Ver personas discapacitadas mendigando por las calles.

Ser diagnosticado con un cáncer terminal.

Ver a un niño llorar por la perdida de su madre.

Perder para siempre a uno de tus mejores amigos.

Pasar el día de tu cumpleaños sin amigos o familiares.

Enterarte del grave accidente de tu hermano estando lejos de casa.

Despedirte de una amiga a la que puede que no vuelvas a ver.

Ser rechazado por tu pareja.

Pasar en soledad los últimos años de tu vida.

Contemplar el dolor de unos amigos por la muerte de su padre.

Darte cuenta de que ya no te quedan amigos cerca.

La muerte de tu querida mascota.

Un barco lleno de refugiados se hunde.

Escuchar una canción que te recuerda a un amigo ya fallecido.

Ver a unos padres destrozados por la pérdida de su hijo.

Pensar en que algún día verás morir a tus padres.

Sentir que estás más solo que nunca.

Enterarte de que tu primo ha perdido una pierna en un accidente.

Ver a una anciana llorando ante la tumba de su marido.

Darte cuenta que ya no te hablas con tus amigos de la infancia.

Separarte de tu pareja porque le tienes cariño pero no la amas.

Ver a un anciano hablar a la tumba de su difunto perro.

Encontrarte con la foto de una amiga recientemente fallecida.

Pensar que si mueres nadie te echará en falta.

Enterarte de que tu cantante favorito ya no podrá volver a cantar.

Asistir a un memorial en recuerdo de las víctimas de un naufragio.

Tener la sensación de que ya no disfrutas con nada.

Sentir que dejaste a la persona que mejor te entiende.

Tener que sacrificar a tu perro para evitar que siga sufriendo.

Ver a tu madre en estado de coma irreversible.

Sentirte vacío y sin ganas de vivir.

Pasar la navidad sin tus recientemente fallecidos padres.

Evocar aquellos momentos familiares que ya nunca volverán.

Perder la ilusión por las cosas de las que siempre has disfrutado.

Leer los mensajes de los familiares de las víctimas de un incendio.

Contemplar cada día la soledad en la que vive tu anciana vecina.

Oír cada mañana el llanto de un niño gravemente enfermo.

Sentir que no eres capaz de hacer feliz a nadie.

Volver al sitio en el que conociste a tu ya fallecida pareja.

Despertarte y comprabar que tu pareja ya nunca dormirá a tu lado.

Vivir de cerca la enfermedad crónica de tu hermana.

Limpiar la caseta de tu querido perro el día después de su muerte.

Contemplar el dolor incurable de un ser querido.

Vivir en un lugar en el que te sientes extraño y aislado.

Sentir que estás muy lejos de tus seres queridos.

Pensar en los planes que ya no podrás realizar con tu pareja.

Estar con amigos y aún así sentirte desmotivado y ausente.
